# Comparative genomics suggests local adaptations in the invasive small hive beetle

**DOI:** 10.1002/ece3.8242

**Published:** 2021-10-26

**Authors:** Yuanzhen Liu, Jan Henkel, Alexis Beaurepaire, Jay D. Evans, Peter Neumann, Qiang Huang

**Affiliations:** ^1^ Vetsuisse Faculty Institute of Bee Health University of Bern Bern Switzerland; ^2^ Vetsuisse Faculty Institute of Genetics University of Bern Bern Switzerland; ^3^ USDA‐ARS Beltsville Bee Research Laboratory Beltsville Maryland USA; ^4^ Agroscope Swiss Bee Research Centre Bern Switzerland; ^5^ Honeybee Research Institute Jiangxi Agricultural University Nanchang China

**Keywords:** invasive species, molecular evolution, natural selection

## Abstract

Invasive species are a major driver of ecological and environmental changes that affect human health, food security, and natural biodiversity. The success and impact of biological invasions depend on adaptations to novel abiotic and biotic selective pressures. However, the molecular mechanisms underlying adaptations in invasive parasitic species are inadequately understood. Small hive beetles, *Aethina tumida*, are parasites of bee nests. Originally endemic to sub‐Saharan Africa, they are now found nearly globally. Here, we investigated the molecular bases of the adaptations to novel environments underlying their invasion routes. Genomes of historic and recent adults *A*. *tumida* from both the endemic and introduced ranges were compared. Analysis of gene–environment association identified 3049 candidate loci located in 874 genes. Functional annotation showed a significant bias toward genes linked to growth and reproduction. One of the genes from the apoptosis pathway encodes an “ecdysone‐related protein,” which is a crucial regulator in controlling body size in response to environmental cues for holometabolous insects during cell death and renewal. Genes whose proteins regulate organ size, ovary activation, and oviposition were also detected. Functions of these enriched pathways parallel behavioral differences between introduced and native *A*. *tumida* populations, which may reflect patterns of local adaptation. The results considerably improve our understanding of the underlying mechanisms and ecological factors driving adaptations of invasive species. Deep functional investigation of these identified loci will help clarify the mechanisms of local adaptation in *A*. *tumida*.

## Graphical abstract

Small hive beetles, Aethina tumida, invasive parasites of pollinating bees have recently invaded multiple continents, across a wide range of climates. The molecular mechanisms underlying the adaptation of this invasive beetle remain poorly understood. Here, the identification of genetic basis, analyses of gene ontology, and pathway enrichment of identified genes provide a mechanistic understanding of a biological invasion.

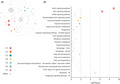

## 1 INTRODUCTION

Biological invasions constitute major ecological and environmental changes that can affect human health, food security, and natural biodiversity (Bradshaw et al., 2016; Pejchar & Mooney, 2009; Renault et al., 2018). Higher production rates of invasive animal, plant, and aquatic species have been well‐documented (Keller et al., 2007, 2011; Wan & Yang, 2016). Indeed, invasive species are often successful in new ecosystems because of release from enemies, parasitism, and resource limits, allowing them to survive, grow, or reproduce at higher rates than in their native ranges (Lockwood et al., 2013; Wan & Yang, 2016). Adaptive evolution of traits that increase survival and reproduction in response to novel selection regimes (abiotic and biotic factors) facilitates the initial establishment and spread of invasive species (Colautti & Lau, 2015), which can influence variation in phenotypes and genotypes of local populations (Colinet et al., 2015; Dillon & Lozier, 2019).

Invasive populations are certain to experience differences in climate, availability of resources, and biotic interactions, as well as disturbance regimes, compared with native populations (Colautti & Lau, 2015; Lee & Gelembiuk, 2008). Prominent examples of rapid evolutionary responses to ambient environments by invasive species include the evolution of *Drosophila* body size across continents with latitudinal climate variation (Huey et al., 2000), the evolutionary changes due to temperature gradient in mosquitoes, *Aedes japonicus japonicus*, after spread into the United States (Egizi et al., 2015), and the phenotypic adaptation of common rabbits, *Oryctolagus cuniculus*, along different climatic regions following invasion into Australia (Williams & Moore, 1989). In special cases of parasitic invasive species, hosts can be also an important factor promoting or mediating the evolution of parasites. Host–parasite coevolution is a strong selective force, which can lead to changes in both genotypes and phenotypes of invasive species. For instance, in the invasive honeybee ectoparasitic mites, *Varroa destructor*, genotypes of parasites infecting mite‐resistant honeybee colonies were shown to significantly differ from susceptible hosts (Beaurepaire et al., 2019). Unlike *V*. *destructor* that parasitizes colonies throughout its life, we know little of how invasive species whose life histories involve both fluctuating external environments and close interaction with hosts evolve. Also, what selection factors are more important, biotic or abiotic factors, during adaptive evolution? A better understanding of ecological and evolutionary processes that underlie successful invasions is important for managing and controlling introduced species, for example, helping to predict the conditions under which invasiveness can be enhanced or suppressed (Olazcuaga et al., 2020).

Invasive small hive beetles (SHBs), *Aethina tumida*, provide an appropriate example to understand this question. SHBs are a worldwide emergent invasive parasite posing considerable threats to global apiculture and wild bees (Neumann et al., 2016). Endemic to sub‐Saharan Africa, SHBs were first detected in 1996 in the United States (Hood, 2000) and have now invaded each habitable continent of the globe (Al Toufailia et al., 2017; Calderón & Ramírez, 2019; Liu et al., 2021; Neumann et al., 2016). SHBs oviposit in nests. Emerging larvae feed on diverse food including honey, pollen, bee brood, dead bees, and conspecific SHBs as well as induced trophallactic feeding from host bees (Gonthier et al., 2019; Neumann et al., 2016). They then exit colonies to pupate in the soil nearby before emerging as adults to invade host colonies (Neumann et al., 2016).

External environments have direct impacts on the adaptation of SHBs. During pupation in the soil (i.e., >75% of their developmental time; de Guzman & Frake, 2007), temperature and humidity constitute severe selective pressures by directly influencing the development, survival, and reproduction of SHBs. Extremely low (≤15°C) or high (≥45°C) temperatures prevent oviposition and egg hatch of SHBs (Annand, 2011), and relative humidity below 34% prevents egg survival (Annand, 2011; Cornelissen et al., 2019; Cuthbertson et al., 2013; Ellis, 2003; de Guzman & Frake, 2007; Rosenkranz et al., 2010; Torto et al., 2010). This may explain the observed seasonal variation in SHB numbers in the native range (rainy vs. dry season) and the generally limited impact of this parasite in semi‐arid and arid environments (Cuthbertson et al., 2013; Ellis, 2003). In contrast, in their invasive range such as North America, SHBs have established populations in regions of cold climates: Maryland, Michigan, and Minnesota in the United States as well as Ontario in Canada (Evans et al., 2003; Neumann et al., 2016). Although pupation success is rather unlikely in the winter, SHBs can overwinter inside the warm winter clusters of bees in temperate regions prior to rebuilding local populations in the spring (Schäfer et al., 2011). The impact of temperature on growth rates and development has been widely documented in arthropods, whereby higher temperatures decrease developmental times more than they increase growth rates, resulting in smaller body sizes in adults (Gardner et al., 2011; Klok & Harrison, 2013; Van der Have & De Jong, 1996; Walters & Hassall, 2006). In SHBs, cooler soil conditions appear to increase pupation time (de Guzman & Frake, 2007; Stedman, 2006), and beetles from the northern United States do trend slightly larger than their southern counterparts (unpublished data).

In addition to environmental parameters, biotic factors may also lead to adaptive changes in SHBs. As parasites and scavengers of bee nests, SHBs are usually considered a minor pest of colonies of African honeybee subspecies (Lundie, 1940). In the invasive ranges, however, they can cause considerable damage to European honeybees (Spiewok et al., 2007), where they can initiate damaging bouts of mass reproduction (Cervancia et al., 2016; Idrissou et al., 2019; Liu et al., 2021; Neumann et al., 2016, 2018). As explained above, the inordinate success of invasive species might result from the release of their co‐evolved natural enemies (Liu & Stiling, 2006), for example, the ant *Pheidole megacephala* that was identified as a key predator of SHB larvae in Kenya (Neumann et al., 2016; Torto et al., 2010). Also, unlike European‐derived honeybee subspecies, African subspecies are more efficient in containing SHB infestation; that is, they are more aggressive, able to trap beetles, and abscond infested colonies only when resources are depleted (Neumann et al., 2016, 2018). The pronounced differences in reproductive capacity between introduced and native populations have likely been driven by the shift to a more permissive host. Besides the above‐mentioned impact of abiotic factors, increased food availability from novel hosts may lead to an increase in beetle size (Ellis, 2003).

Here, we present evidence for genetic mechanisms that underlie global invasion and local adaptation in SHBs. We hypothesize that genes associated with reproduction and body size might be affected due to selection following invasion and adaptation to new environments. To test this, we sequenced individuals from seven representative groups across the native and invasive regions, including two previously suggested ancestor populations to the introduced US populations (Evans et al., 2000; Idrissou et al., 2019). The selection signatures and candidate genes associated with local adaptation in response to novel environments were identified by comparing the genomes of the introduced US population with its African ancestor populations using analysis of gene–environment association.

## 2 MATERIALS AND METHODS

### 2.1 Sampling and DNA extraction

**FIGURE 1:**
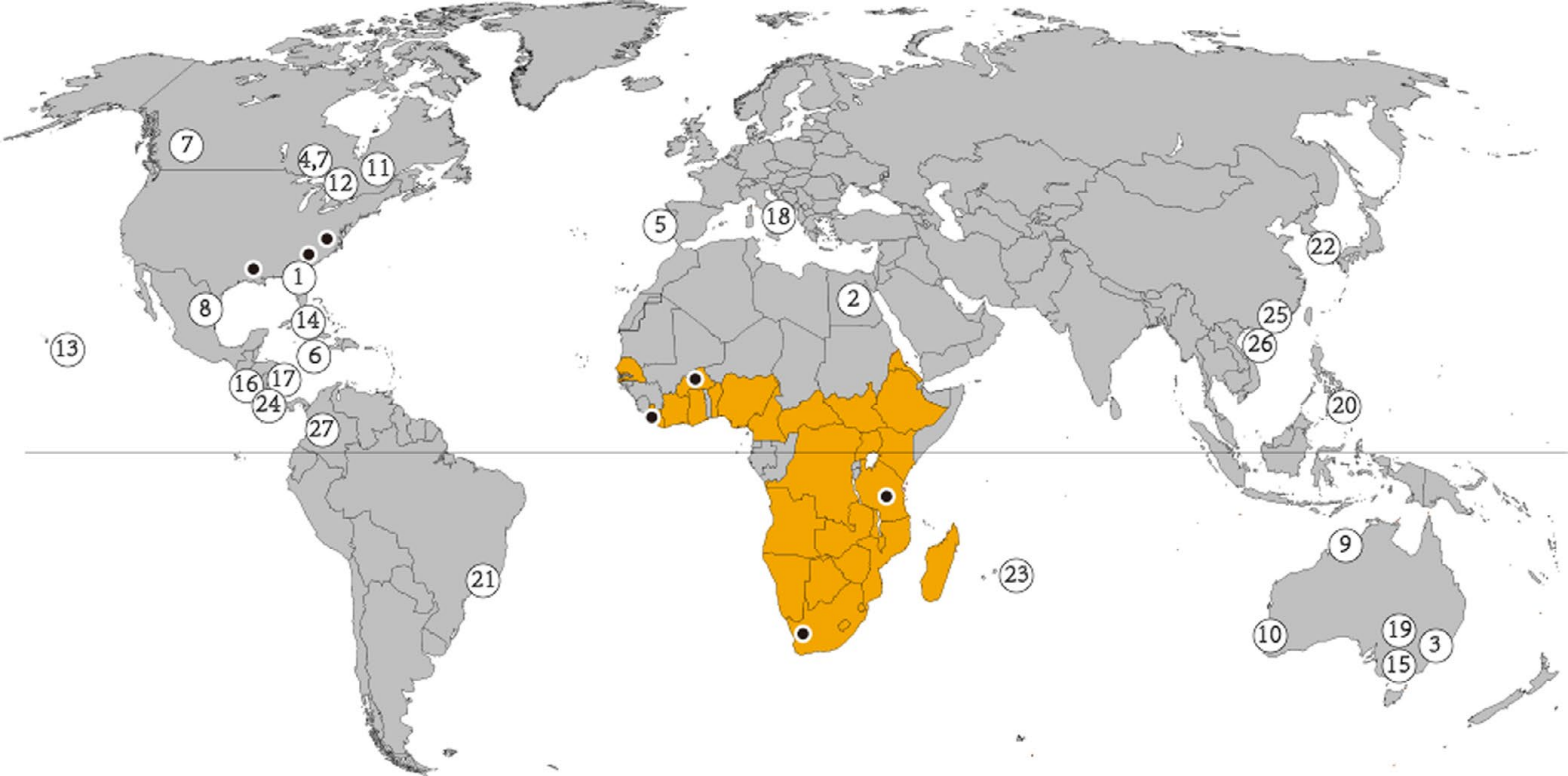
Global distribution and sampling of the adult small hive beetles, *Aethina tumida*. Orange areas indicate records of *A*. *tumida* in the native range in sub‐Saharan Africa. Black dots indicate the sampling locations in the United States (Maryland, South Carolina, and Louisiana) and sub‐Saharan Africa (Liberia, Burkina Faso, Tanzania, and South Africa). The incremental numbers in the circles denote the chronological detection of *A*. *tumida* invasions globally up to December 2020 (Idrissou, Huang, et al., 2019; León, 2021; Liu et al., 2021)

Since previous studies suggested that Tanzania and/or South Africa were the most likely source populations of beetles introduced into the United States (Evans et al., 2000; Idrissou, Huang, et al., 2019), we sampled beetles between 2015 and 2018 from infested stationary *Apis mellifera* colonies in the native range of the parasite in sub‐Saharan Africa (Neumann et al., 2013) and the invasive range in the United States. The collected samples covered the southern and northern limits and the middle of the native range, including South Africa, Burkina Faso, Liberia, and Tanzania, respectively, as well as Louisiana and Maryland in the United States (*N* = 8–12; Figure 1). Historic samples collected in 1999 from an incipient population in South Carolina, United States, were also included (*N* = 7). To ensure taxonomic status, samples were first confirmed as SHB using morphometrics (Neumann et al., 2013). Then, their COI gene sequences were aligned with those of already identified SHB (GenBank accession MK025192.1) (Idrissou, Huang, et al., 2019). High‐quality DNAs of 64 recent samples were extracted using the NucleoSpin^®^ Tissue Kit and fragmented DNA of four historical samples (low quality) using a phenol–chloroform extraction protocol (Barnett & Larson, 2012) following the manufacturer's instruction. TruSeq^®^ DNA PCR‐Free libraries for each population (2 × 150 bp paired‐end reads) were prepared for whole‐genome sequencing with the Illumina NovaSeq S4 and NovaSeq 6000 (only for Tanzania samples) sequencers following standard procedures.

### 2.2 Quality control and read mapping

We estimated the quality of the raw sequencing data of SHB samples using FastQC v0.11.5 (https://www.bioinformatics.babraham.ac.uk/projects/fastqc/). Low‐quality bases and adaptors, as well as short reads, were removed and trimmed with fastp v0.12.5 (settings: ‐q 15 ‐l 20; Chen et al., 2018). The same processes were applied to the five publicly available sequence sets from the Asian longhorned beetle, *Anoplophora glabripennis* (accession number: PRJNA167479; McKenna et al., 2016), that were used as an out‐group. The obtained clean reads were mapped to the SHB reference genome using Burrows‐Wheeler Aligner (bwa mem) v0.7.13 with default parameters and “M” flag to mark the shorter alignments as secondary (Li & Durbin, 2009).

### 2.3 Variant calling and genotyping

Single nucleotide polymorphisms (SNPs) were called following the recommended variant calling steps of the Genome Analysis Toolkit HaplotypeCaller v3.8 (McKenna et al., 2010). Due to no known SNP data for SHB, a moderate hard‐filtering of the SNPs was performed (Auwera et al., 2013).

### 2.4 Analyses of population relationships

Principal component analyses (PCA) based on all bi‐allelic SNPs among the 68 SHB individuals were performed using the software PLINK v1.9 (Purcell et al., 2007) to identify population structure across the geographical location. The first three significant components were plotted. Linkage disequilibrium (LD) was estimated to reflect recombination history for each SHB population with program PopLDdecay (Zhang et al., 2019) by correlation coefficients (*r* ^2^) between SNP pairs (missing call rate <0.1) using software PLINK (Purcell et al., 2007). A neighbor‐joining (NJ, *p*‐distance, pairwise deletion) tree was constructed using MEGA v7.0 (Kumar et al., 2016) with the sequences consisting of the whole set of SNPs to present the genetic relationships within and among populations. Historical samples were excluded because of too many gaps. Bootstrap analyses with 1000 iterations were used to assess the branch reliability. Population structure was further analyzed in the program Admixture v1.3.0 (Alexander et al., 2009) with the assumed number of ancestral populations (*K*) from 2 to 7. Both analyses were conducted by using all non‐admixed samples of each group. Cross‐validation errors were used to estimate a reasonable value of *K* for model fitness. Based on the results of PCA, NJ, and structure analyses, we separated individuals that had apparent stratification within the same population into subpopulations for the subsequent analyses. SNPs with a missing call rate <0.9 were applied to compute the pairwise kinship coefficients as well as the population fixation statistic (*F*_ST_) (20 kb windows sliding in 10 kb steps) using VCFtools v0.1.15 (Danecek et al., 2011).

### 2.5 Gene–environment association (GEA)

We performed a GEA study using the SHB whole‐genome sequencing data and bioclimatic variables to detect genomic signatures of adaptation to climate in SHBs between the native source population and introduced one. The genomic data consisted of 11,383,788 genetic variants (SNPs) genotyped for 30 individuals from an introduced US (Maryland) population and its source (ancestor) populations in the native range—South Africa and Tanzania, according to the relationships of these populations. In this way, we can avoid cofounded mutations because of evolutionary history. SNPs with minor allele frequency <.05, missing data, and *r* ^2^ > .4 (Figure S1) were filtered out to generate a matrix, which contained 157,556 informative SNPs (Danecek et al., 2011, 2021; Frichot & François, 2015). The publicly available data of bioclimatic variables (~1 km^2^) were downloaded from the WorldClim database (Fick & Hijmans, 2017) using geographical coordinates of 3–4 sites (Table S1) around each geographical location of sampling. Considering the environmental variables most likely causing strong selection, we summarized the minimal mean temperature and the minimum mean precipitation of the driest month using PCA, and the first component was used as a new variable for each population (Frichot et al., 2013).

The program LFMM (latent factor mixed models) was used to find the correlation with environmental gradient after the number of latent factors *K* (individual admixture coefficients or ancestral populations) was estimated using snmf() function in LEA (Caye et al., 2019; Frichot & François, 2015). Following the manual of LFMM, six runs with settings (‐n 30 ‐L 157556 ‐D 1 ‐K 2 ‐p 2 ‐i 500000 ‐b 100000) were performed to re‐adjust the *p*‐values to increase the power of the LFMM test statistic (Caye et al., 2019). The candidate loci were adjusted for a false discovery rate (FDR) of 10%.

### 2.6 Functional annotation

To assess the potential functionality of genes harboring these candidate loci, SNPs across populations of South Africa, Tanzania, and the United States (Maryland) were annotated by a homology‐based method with the GFF gene models of *A*. *tumida* (Evans et al., 2018) via SnpEff (snpEff_v4_5covid19_core) eliminating any upstream and downstream effects by using "‐ud 0" (Cingolani et al., 2012). The accession numbers of the identified protein‐coding genes in SHBs were used to retrieve corresponding protein sequences that were aligned to the model beetle species *Tribolium castaneum* protein sequences (Tcas 5.3; Poelchau et al., 2015) using BLASTP in BLAST+2.10.1 (Camacho et al., 2009). To gain insight into potential biological functions that are likely to be involved in adaptive evolution toward diverse climates, the mapped *T*. *castaneum* protein sequences of these genes were submitted to Panther 15.0 (Thomas et al., 2003) for gene ontology (GO). The protein sequences of the identified SHB genes were submitted to the webserver of Kobas 3.0 (Xie et al., 2011) for Kyoto Encyclopedia of Genes and Genomes (KEGG) pathway enrichment analysis, with the *T*. *castaneum* genes as a reference gene set. A cutoff of 0.05 for FDR (Benjamini and Hochberg) was used to determine the significant level of enriched pathways (Thomas et al., 2003).

### 2.7 Data analysis and visualization

The phylogenetic tree was visualized in FigTree v1.4.4 (https://github.com/rambaut/figtree/releases/download). In R (R Core Team, 2013), the PCA and Manhattan graphs were plotted using “plot ()” and “qqman” (Turner, 2014), respectively.

## 3 RESULTS

### 3.1 Data processing

The 64 recent samples generated an average number of 70.6 million paired‐end reads of 150 bp read length, and on average, 22.98 million such reads were obtained for the four historical specimens (Table S2). Analyzing these 68 samples together with five *Anoplophora glabripennis* individuals yielded a set of 11,465,251 SNPs after a moderate hard‐filtering step (about 70% variants remaining).

### 3.2 Population structure

**FIGURE 2:**
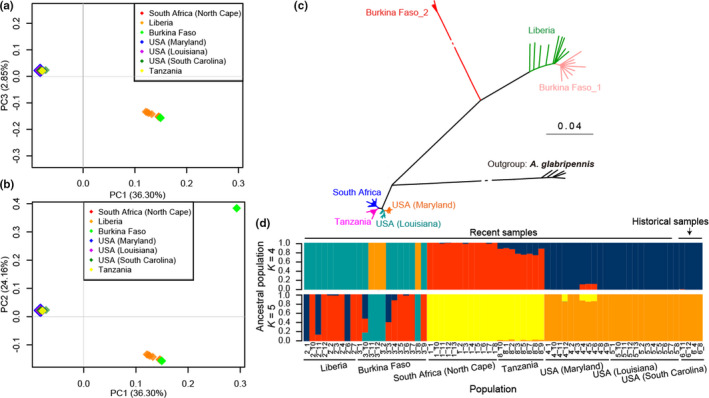
Population structure. (a–b) Two‐way PCA plots of the first two components (a: PC1 and PC3; b: PC1 and PC2) of the seven small hive beetles, *A*. *tumida*, populations. Each *A*. *tumida* population is represented in a color. (c), Neighbor‐joining phylogenetic tree of *A*. *tumida* populations derived from 1000 bootstrap replicates using program MEGA. Historical samples were excluded due to too many gaps. The tree was rooted with *A*. *glabripennis*. The scale bar indicates the evolutionary distances estimated by the *p*‐distance model. (d), Admixture of the *A*. *tumida* populations. The sample types were on the top (recent or historical samples), and geographic locations are at the bottom. The partition of each colored segment indicates the percentage of each sample's genome from *K* = 4 to 5 ancestral populations, in which *K* = 4 is the best fit model based on the cross‐validation error estimate using program Admixture

The PCA analysis showed that SHB samples formed three major clusters, among which the Burkinabe population had two apparent stratifications: one similar to the Liberian population and one independent cluster. The remaining SHB samples from South Africa, Tanzania, and the United States clustered together (Figure 2a,b). These relations were further reflected by the neighbor‐joining (NJ) tree (Figure 2c). These results were also consistent with the assignment by the software Admixture (Figure 2d). Changing the number of presumed ancestral populations (*K*) disclosed genetically distinct clusters that mirrored both geographic proximity and gene flow. When *K* = 4 (determined as the best fit model), a similar structure was found between the Liberian and Burkinabe populations, as well as for the South African, Tanzanian, and American populations (Figure 2d). These results supported that South Africa and Tanzania populations were genetically close, and South Africa and Tanzania populations were reconfirmed as the ancestor populations of the US one.

*F*_ST_ values (Figure S2) between populations showed little genetic differentiation between the United States (South Carolina) and South Africa/Tanzania (0.0149 and 0.0089, respectively) and moderate genetic differentiation between the US populations of Maryland and Louisiana and that of South African and Tanzanian populations (0.1476–0.1772).

### 3.3 Identification of outlier loci

**FIGURE 3:**
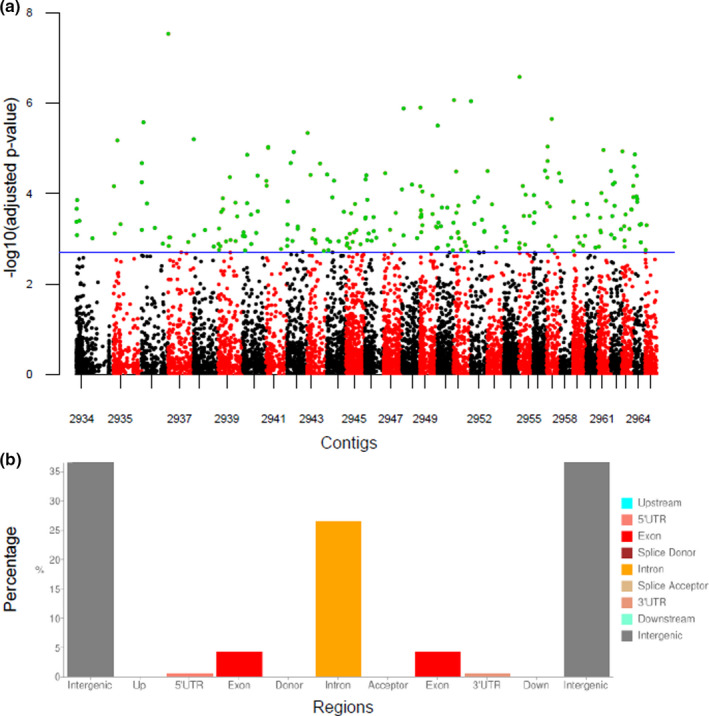
Outlier SNPs (false discovery rate = 10%) and the distributions of variants in genomic regions. (a) Distribution of outlier SNPs on the first 32 contigs. The SNPs for each contig are distinguished by black and red dots, whereas the outlier dots, −log10(adjusted *p*‐value) > 2.713, are highlighted in green. The names of contigs on the *x*‐axis are abbreviated to four digits, for example, the first one referring “NW_017852934.1”. (b) The distribution of variants in genomic regions excluding upstream and downstream effects of each SNP

The individual admixture coefficients among South Africa, Tanzania, and the United States (Maryland) were estimated as *K* = 2 (Figure S3a–b), and a list of 3049 candidate loci out of 157,556 variants (1.94%) associated with environmental gradients was obtained with an expected FDR level of 10% (Figure 3a). The majority of these loci were placed in intergenic and intron regions (Figure 3b), which involved 874 genes including nine pseudogenes and 10 genes of predicted non‐protein‐coding transcripts.

### 3.4 Function classification and pathway enrichment

**FIGURE 4:**
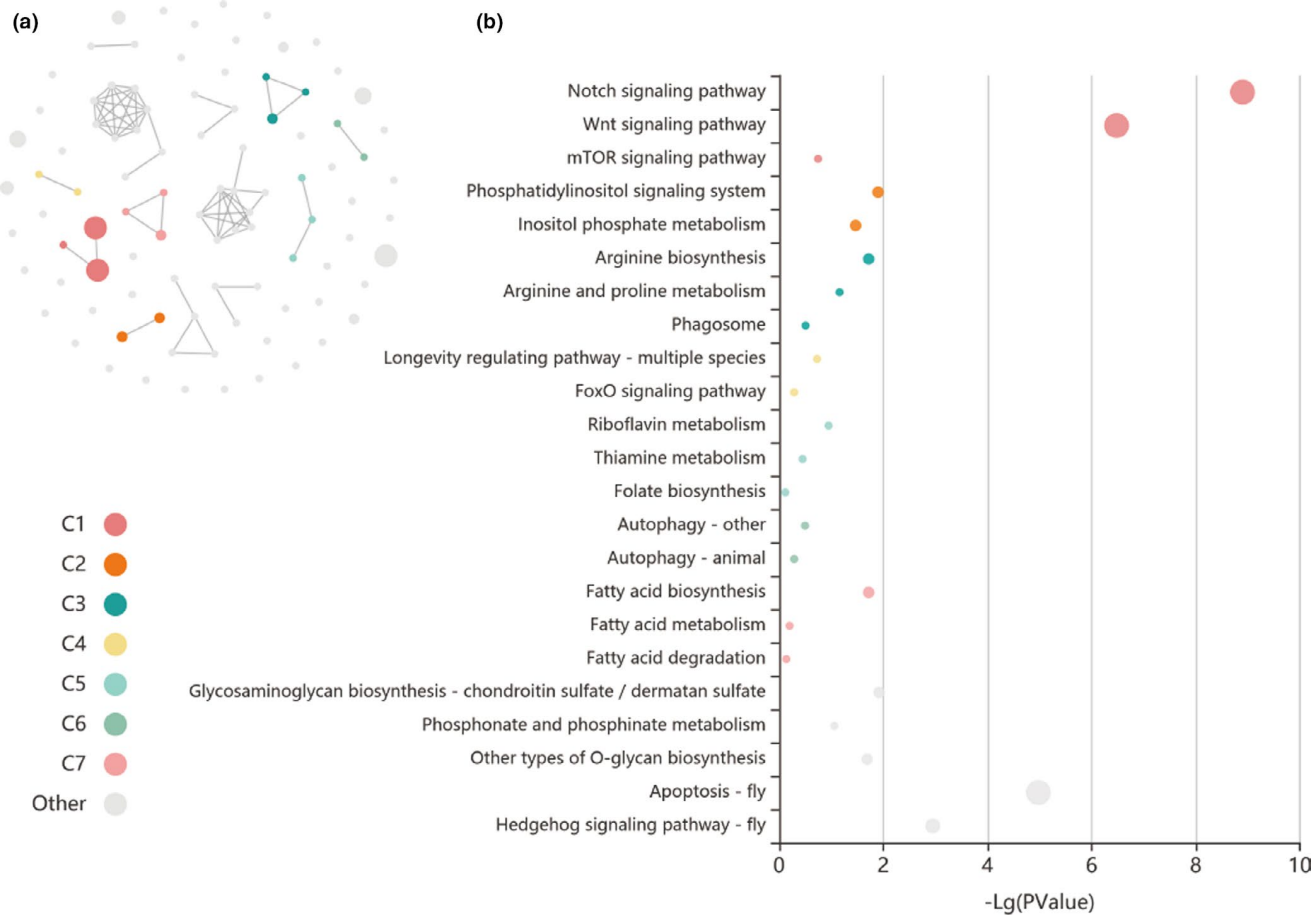
Enriched KEGG terms based on Kobas database. (a) Circular network of the enriched gene terms. Each node represents an enriched term, and the node color represents different clusters (C1–C7 and other); the node size represents six levels of enriched *p*‐value, node size from small to large: [0.05,1], [0.01,0.05), [0.001,0.01), [0.0001,0.001), [1e−10,0.0001), [0,1e−10); the edge represents correlations larger than 0.05. (b) Enriched functions of candidate genes. Each bubble represents an enriched function, and the size of the bubble represents six levels of enriched *p*‐value as explained above. The colors of the nodes are the same as the colors in the circular network, which represent different clusters (C1–C7 and others). For each cluster, if there are more than five terms, the top five with the highest enrich ratio are displayed

The mapped protein sequences of 737 unique homologs in *T*. *castaneum* were predominantly involved in molecular function such as “binding,” “catalytic activity,” and “molecular function regulator” (Figure S4a), as well as biological processes including “cellular process,” “biological regulation,” and “metabolic process” (Figure S4b). The enrichment of pathways further suggested their interactions among these genes. Six pathways were significantly enriched (corrected *p*‐values < .05) with four displayed in Figure 4a–b and another two “Hippo signaling pathway” and “Neuroactive ligand‐receptor interaction” in Table S3. Two top enriched pathways, “Notch signaling pathway” and “Wnt signaling pathway,” are shown to interplay. Forty‐nine genes associated with these pathways were summarized in Table S4, in which some were involved in multiple pathways.

## 4 DISCUSSION

In this study, we characterized the relationships of seven representative SHB populations across native and introduced ranges and the genomic response to local adaptation. We verified the ancestor populations of the SHBs introduced into the United States. Our association analysis between loci and environmental gradients using LFMM led to insights into the evolutionary history and genetic basis of local adaptation between the introduced population and its ancestor populations. Analysis of gene–environment association identified 3049 genome sites located in 874 genes. Functional annotation showed that several top enriched pathways reflected by 49 genes were closely related to growth and reproduction, which seemed to parallel the behavioral differences between introduced and native SHB populations and may reflect their local adaptations. These results supported our hypothesis that genes associated with reproduction and body size have been involved in the adaptive evolution of invasive SHBs.

By comparing the samples of introduced populations with their ancestor populations, we found little differentiation across the genome and a majority of variants (63%) that were non‐coding transcript variants located in intergenic and intron regions. However, these outlier variants by GEA analysis indicated that allele frequencies differed strikingly between the introduced and their ancestral populations. The designed comparison in combination with the LFMM method that considered confounding factors and genotype–environmental analyses allowed us to identify reliable polygenetic signatures of recent adaptation, because the random drifts and the cofounding factors influence the correlation with environment gradient and this method had more power than the *F*_ST_ outlier tests (Caye et al., 2019; Welles & Dlugosch, 2019). As for the environmental variables used in this study, maximum temperatures were not included because they were similar across populations and would result in an identical first principal component which was not helpful to screen selection signatures of environmental adaptation across genomes. We were aware that most variants would be located outside coding genes as discovered by the extensive genome‐wide association studies, and most often one tended to focus on those on protein‐coding exons (Giral et al., 2018; Maurano et al., 2012).

An increasing number of studies have shown that phenotypic variability could be influenced by genetic variations outside of coding genes acting as regulatory elements such as enhancers and/or involving in transcriptional regulation (Giral et al., 2018; Perenthaler et al., 2019). Such variants associated with diseases and specific traits have been reported in humans and insects (Giral et al., 2018; Kocher et al., 2018; Zhao et al., 2017). Indeed, many differentiated SNPs found in the introns or untranslated regions faced a selective sweep in the introduced population in contrast to a great deal of standing genetic variation in native populations (Perenthaler et al., 2019). Recent genomic studies provide increasing evidence for the role of standing genetic variation as the predominant source in fast local adaptation of invasive species, as opposed to new mutations (Lai et al., 2019; Prentis et al., 2008), because beneficial alleles are often immediately available from standing variation and usually start at higher frequencies than the newly emerged ones, as well as enable beneficial alleles to spread and fix within the population rapidly when environmental changes occur (Barrett & Schluter, 2008; Hermisson & Pennings, 2017). Similarly, our analyses showed that multiple loci are likely linked to the local adaptation or differentiation of SHBs between native and introduced populations, which had a handful of intronic SNPs with extreme allele frequency difference ~20 years post‐invasion in the United States (Hood, 2000).

The functions of genes identified to be associated with local adaptation in this study seemed to reflect the current obvious differences of SHBs found in the United States and native range, for example, in body size and fertility. Two of the enriched pathways, the Apoptosis and the Hippo signaling pathways, appeared to play a key role in adaptive growth. In insects, genes that encode ecdysone‐related proteins such as “ecdysone‐induced protein 74EF‐like” and “ecdysone receptor‐like” that participate in apoptosis are crucial regulators in controlling body size in response to environmental cues especially for holometabolous insects during cell death and renewal (Nijhout et al., 2014). Parts of this mechanism involve the interplay between two hormones, the juvenile hormone (JH) and the molting hormone ecdysone (Riddiford, 2012; Riddiford et al., 2003). A study in the insect, *Manduca sexta*, showed that thermal effects on developmental duration were due to changes in the length of time from critical weight to molting, indicating that the removal of JH or the secretion of ecdysone might be strongly temperature‐sensitive (Davidowitz et al., 2004). Within insect species, generally, adults of populations from higher altitudes or latitudes are generally larger than those from lower altitudes or latitudes (Arnett & Gotelli, 1999; Hernández‐L et al., 2010; James et al., 1997). Despite lacking systematic studies that compare the biometrics of adult SHBs between the introduced and ancestor populations, the body size of current adult SHBs of introduced populations in northern regions of the United States tends to be larger than for adults of its invasion in southern regions, analogous to ancestors in South Africa or Tanzania, that is, when we compared the northern US wild adult SHBs from Maryland (male body length: 5.7 ± 0.3 mm, female body length: 6.3 ± 0.4 mm, unpublished data) with historical data of mean values of two southern states: South Carolina and Georgia (male body length: 5.5 ± 0.01 mm, female body length: 5.7 ± 0.02 mm; Ellis et al., 2002). However, besides temperature, nutrition can alter growth rates as well (Nijhout et al., 2014; Robertson, 1963). Thus, systematic morphometric comparisons of samples adapted in different latitudes and reared in the same environment will be indispensable to provide evidence for this regard. A gene coding the “transcriptional coactivator YAP1‐A” is one of the key and major effectors of the Hippo pathway, as suggested by its name the major and best‐characterized function is its transcription coactivator activity (Ma et al., 2019). This pathway is an evolutionarily conserved signaling cascade regulating numerous biological processes, including cell growth, organ size control, and regeneration (Dong et al., 2007; Ma et al., 2019). The deletion of YAP in mice suppressed the overgrowth phenotypes (Zhou et al., 2011). Notably, some gene members of this pathway were highly enriched for cold‐adapted honeybees, *A*. *mellifera sinisxinyuan* n.ssp (Chen et al., 2016). This pathway has also been suggested to be involved in thermal tolerance by upregulation (Cheng et al., 2020). Taken together, the control of growth and related physiological responses can be important aspects for SHBs during adaptation to novel habitats.

Another interesting characteristic is that the roles of other enriched pathways paralleled the behavioral differences between introduced and native populations. SHB mass production of thousands of larvae is often observed in European‐derived honeybee colonies in the introduced areas, while being very rare in colonies of African honeybee subspecies, *A*. *m*. *scutellata* and *A*. *m*. *capensis* (Idrissou, Huang, et al., 2019; Neumann et al., 2018). This remarkable difference apparently reflects the high degree of ovary activation, reproduction capacity, and evolutionary divergence, perhaps in response to novel habitats with not only different climates but enemy release as well as host shifts with less constraint (Neumann et al., 2016). Wnt and Notch are ancient and conserved cell signaling pathways across animals, which interplay and regulate gene expression in developments including posterior growth, axis patterning in embryogenesis, control of the sexually dimorphic development of reproductive organs, oogenesis, and reproduction (Chesebro et al., 2013; Duncan et al., 2016; Guruharsha et al., 2012; Hayward et al., 2005; Martin & Kimelman, 2009; Murat et al., 2010). One specific gene coding the “WNT1‐inducible‐signaling pathway protein 1” had the largest number of outlier SNPs (Table S4), which may indicate the strong selection on the Wnt signaling pathway. Intriguingly, recent studies have shown that the Notch cell signaling has a functional role in repressing the development of honeybee‐worker ovaries and the chemical inhibitor of Notch signaling increased the proportion of bees with active ovaries (Duncan et al., 2016). Also, the analyses of differentially expressed RNAs in different phases of the queens revealed a few significantly enriched pathways including Notch and Wnt that are closely related to oviposition (Chen et al., 2017). The two studies demonstrated that the queen pheromone is essentially the causal factor associated with the Notch receptor degradation and loss of Notch signaling in honeybees. The use or impact of host‐related chemicals such as honeybee alarm pheromones by SHBs has been reported (Torto et al., 2007). It is worth investigating whether the mass reproduction of SHBs in abandoned colonies or colonies of defense fails is resulted from lacking or weak queen pheromone that inhabits SHBs’ ovary activation and development.

Although these pathways discussed above do not seem to directly correlate with adaptation to different climate regimes, the genes might be evolutionarily convergent due to selection reflecting diverse ecological factors. Selection also affects the frequency of linked variants because of hitchhiking, which generates genomic divergence between populations experiencing different environments (Garner et al., 2018; Montero‐Mendieta et al., 2019; Sabeti et al., 2007). Nevertheless, our results support our hypothesis that genes associated with reproduction and body size are involved in the adaptive evolution of SHBs.

## 5 CONCLUSION

We here explored genetic bases for the adaptation of invasive species to novel environments. The identified genes may directly contribute to the invasion success of a destructive bee parasite (Idrissou, Huang, et al., 2019; Neumann et al., 2016). Overall, this enhanced genomic knowledge has improved our understanding of the underlying mechanisms and ecological factors driving adaptation of invasive species. In addition, this data set will perhaps constitute a start for future evolutionary genomics of invasive species, enabling to tackle further key factors explaining their successful adaptation to novel environments, ranging from enemy release (Liu & Stiling, 2006; Torchin et al., 2003) to host shifts (Singh et al., 2020; Woolhouse et al., 2005).

## CONFLICT OF INTEREST

The authors declare no conflict of interest.

## AUTHOR CONTRIBUTION

**Yuanzhen Liu:** Conceptualization (lead); Data curation (lead); Formal analysis (lead); Investigation (lead); Methodology (lead); Resources (supporting); Software (lead); Validation (lead); Visualization (lead); Writing‐original draft (lead); Writing‐review & editing (equal). **Jan Henkel:** Formal analysis (supporting); Writing‐review & editing (supporting). **Alexis Beaurepaire:** Writing‐review & editing (equal). **Jay Daniel Evans:** Methodology (equal); Resources (supporting); Supervision (equal); Writing‐review & editing (supporting). **Peter Neumann:** Conceptualization (equal); Funding acquisition (lead); Investigation (supporting); Project administration (lead); Resources (lead); Supervision (supporting); Writing‐review & editing (supporting). **Qiang Huang:** Conceptualization (equal); Funding acquisition (lead); Investigation (supporting); Methodology (supporting); Supervision (equal); Writing‐review & editing (equal).

## Supporting information

Fig S1‐S4

Table S1, S3‐S4

Table S2

## Data Availability

Sequences associated with this study have been deposited at the NCBI Sequence Read Archive: https://trace.ncbi.nlm.nih.gov/Traces/sra/?study=SRP305033 under project No. PRJNA698343.
