# Supplementary material for: Comparative genomics suggests local adaptations in the invasive small hive beetle
Source: Ecol Evol. 2021 Oct 26;11(22):15780–91. doi: 10.1002/ece3.8242 (PMC8601931; doi:10.1002/ece3.8242)
Supplement: Supplementary file 1 — Fig S1‐S4 [file ECE3-11-15780-s003.pdf]

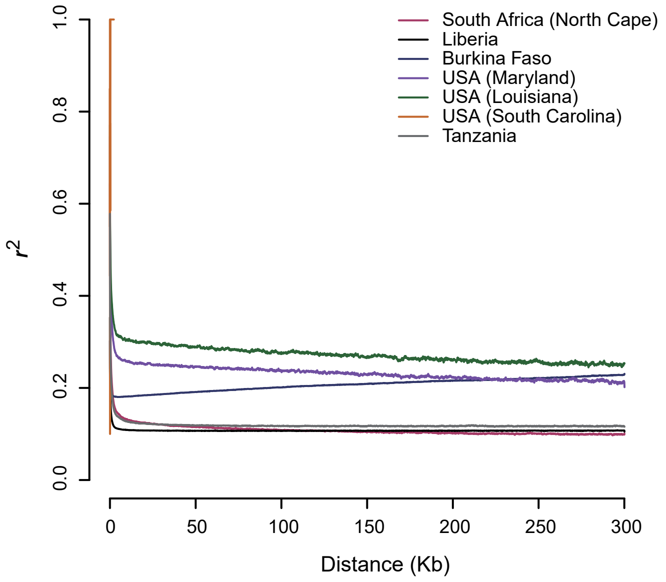

Figure S.1 Linkage disequilibrium decay analysis of these small hive beetle populations. The curves indicate the non-linear regression of r^2 onto the physical distance in base pairs.

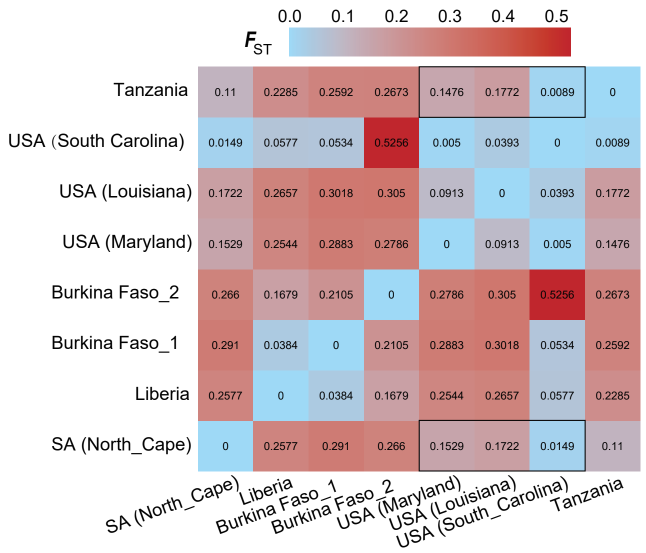

Figure S.2 Mean genetic differentiation (F_{ST}) among populations based on F_{ST} values of all SNPs, calculated in 20 kb sliding window in 10 kb steps using program VCFtools. The color range presents the F_{ST} values, and the black rectangles mark the F_{ST} values between South African or Tanzanian population and three USA populations, respectively.

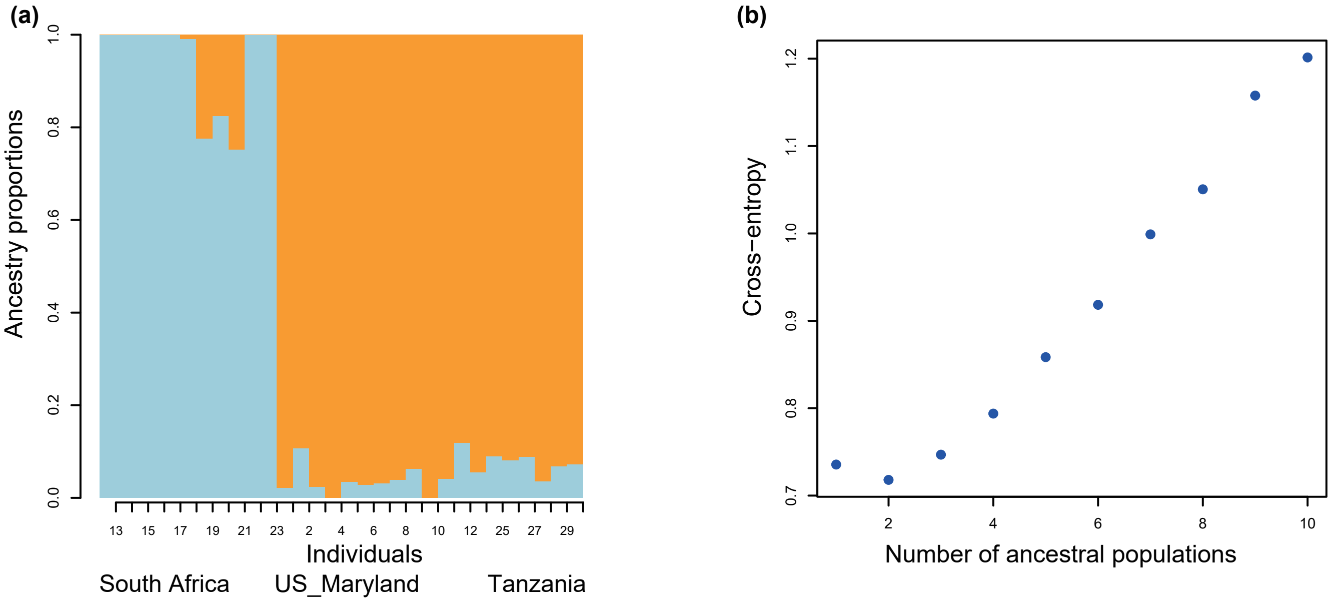

Figure S.3 Estimating the number of ancestry population. **(a)** Barplot of ancestry coefficients obtained from snmf() when ***K*** = 2 in LEA program. **(b)** Value of the cross-entropy criterion as the number of population in LFMM2 program.

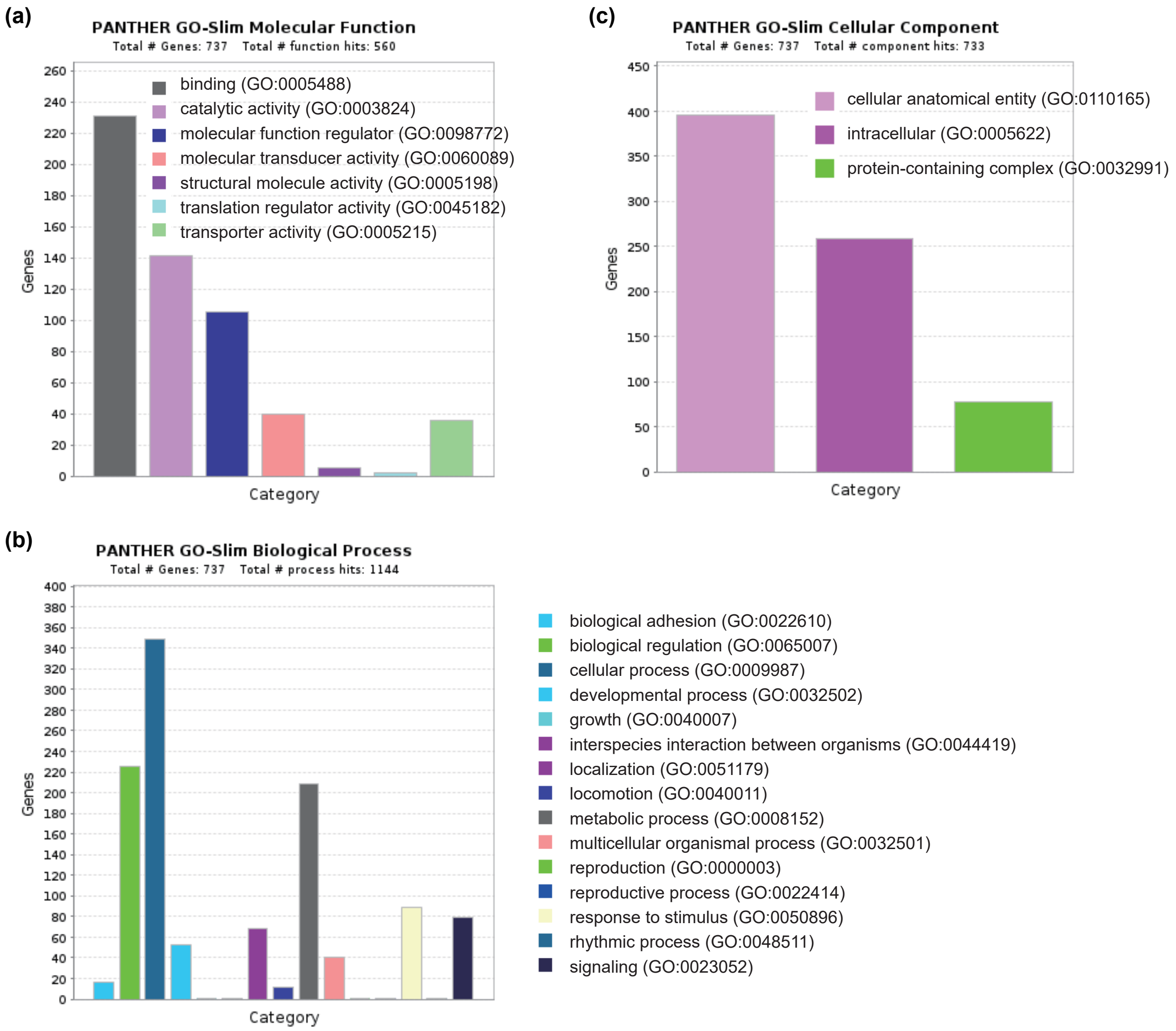

Figure S.4 Classification of gene ontology (GO) terms for the candidate genes. These GO terms were obtained by submitting the mapped protein sequences of homologues in *Tribolium castaneum* to Panther database.
