# Supplementary material for: Comparative genomics suggests local adaptations in the invasive small hive beetle
Source: Ecol Evol. 2021 Oct 26;11(22):15780–91. doi: 10.1002/ece3.8242 (PMC8601931; doi:10.1002/ece3.8242)
Supplement: Supplementary file 2 — Table S1, S3‐S4 [file ECE3-11-15780-s001.pdf]

Table S.1 Geographical coordinates of sampling locations

Location	Latitude	Longitude	Mean minimal temperature (10X°C)	Mean minimal precipitation (mm)	Principal component 1
South Africa (North Cape)	-28.857853	20.108158	24.25	3.25	-0.57735
	-28.896334	20.646244			
	-29.64385	21.624272			
	-29.662945	20.481694			
Tanzania (Arusha, Kilimanjaro)	-3.399442	36.655519	124	14.75	-0.57735
	-3.185005	37.161319			
	-3.314134	37.094341			
	-3.445551	36.75945			
US (Maryland-Baltimore, Lutherville)	39.026173	-76.923531	-49	75.66666667	0.57735
	39.402082	-76.666953			
	39.322536	-76.649614			

Table S.3 Significantly enriched KEGG pathways

Term	Database	ID	Input gene No.	Total gene No.	*P*-value	Corrected *P*-value
Notch signaling pathway	KEGG PATHWAY	tca04330	17	24	1.25E-09	1.36E-07
Wnt signaling pathway	KEGG PATHWAY	tca04310	25	86	3.31E-07	1.80E-05
Apoptosis - fly	KEGG PATHWAY	tca04214	16	48	1.05E-05	3.81E-04
Hippo signaling pathway - fly	KEGG PATHWAY	tca04391	15	54	1.13E-04	3.07E-03
Neuroactive ligand-receptor interaction	KEGG PATHWAY	tca04080	16	64	1.91E-04	4.15E-03
Hedgehog signaling pathway - fly	KEGG PATHWAY	tca04341	9	28	1.12E-03	2.04E-02

Table S.4 Summary of genes associated with six significantly enriched KEGG pathways

Pathway term	Contig	Gene ID*	Product	No. of outlier SNPs
Wnt signaling	NW_017853403.1	LOC109603270	segment polarity protein dishevelled homolog DVL-3	1
	NW_017853000.1	LOC109595221	serine/threonine-protein phosphatase 2B catalytic subunit 3-like	1
	NW_017853676.1	LOC109605320	division abnormally delayed protein-like	1
	NW_017853561.1	LOC109604690	protein groucho-like	1
	NW_017853824.1	LOC109605796	C-terminal-binding protein	1
	NW_017853134.1	LOC109599437	axin-1	1
	NW_017853112.1	LOC109598845	WNT1-inducible-signaling pathway protein 1	4
	NW_017853152.1	LOC109599876	protein groucho-1-like	2
	NW_017853945.1	LOC109606177	1-phosphatidylinositol 4,5-bisphosphate phosphodiesterase classes I and II-like	1
	NW_017852964.1	LOC109609363	protein prickle-like	4
	NW_017853008.1	LOC109595558	protein pangolin, isoforms A/H/I/S (LOC109595558)	2
	NW_017853794.1	LOC109605682	tyrosine-protein kinase transmembrane receptor Ror-like	1
	NW_017852972.1	LOC109593930	serine/threonine-protein kinase NLK-like	1
Notch signaling	NW_017853403.1	LOC109603270	segment polarity protein dishevelled homolog DVL-3	1
	NW_017853152.1	LOC109599876	protein groucho-1-like	2
	NW_017853824.1	LOC109605796	C-terminal-binding protein	1
	NW_017853085.1	LOC109598146	fringe glycosyltransferase	1
	NW_017853456.1	LOC109603854	protein numb-like	1
	NW_017853004.1	LOC109595403	neurogenic locus Notch protein-like	1
	NW_017853429.1	LOC109603518	histone acetyltransferase KAT2A	1
	NW_017853561.1	LOC109604690	protein groucho-like	1
Apoptosis	NW_017853179.1	LOC109600475	mushroom body large-type Kenyon cell-specific protein 1	2
	NW_017853575.1	LOC109604748	ecdysone-induced protein 74EF-like	1
	NW_017853233.1	LOC109601268	ecdysone receptor-like	1
	NW_017853331.1	LOC109602307	tumor necrosis factor receptor superfamily member wengen	2
Hippo signaling	NW_017853410.1	LOC109603316	uncharacterized LOC109603316	1
	NW_017853280.1	LOC109601791	palmitoyltransferase ZDHHC18	2
	NW_017853247.1	LOC109601412	disks large 1 tumor suppressor protein-like	3
	NW_017853267.1	LOC109601635	transcriptional coactivator YAP1-A	3
	NW_017852944.1	LOC109602865	atypical protein kinase C-like	1
	NW_017853331.1	LOC109602307	tumor necrosis factor receptor superfamily member wengen	2
	NW_017853676.1	LOC109605320	division abnormally delayed protein-like	1
	NW_017853934.1	LOC109606151	actin, acrosomal process isoform-like	1
	NW_017853504.1	LOC109604222	protein dachsous-like	1
	NW_017853640.1	LOC109605155	protein spitz-like	1
	NW_017853501.1	LOC109604211	arginine-glutamic acid dipeptide repeats protein	1
Neuroactive ligand-receptor interaction	NW_017853061.1	LOC109597325	metabotropic glutamate receptor 4-like	1
	NW_017853014.1	LOC109595741	tachykinin-like peptides receptor 86C	1
	NW_017853338.1	LOC109602380	parathyroid hormone/parathyroid hormone-related peptide receptor-like	1
	NW_017853758.1	LOC109605562	glutamate receptor ionotropic, NMDA 2B-like	1
	NW_017852966.1	LOC109609436	metabotropic glutamate receptor-like	1
	NW_017853151.1	LOC109599863	neuromedin-U receptor 2-like	1
	NW_017853689.1	LOC109605386	calcitonin gene-related peptide type 1 receptor-like	1
	NW_017853380.1	LOC109603003	probable muscarinic acetylcholine receptor gar-2	2
	NW_017852943.1	LOC109602514	gonadotropin-releasing hormone II receptor-like	1
	NW_017854898.1	LOC109607544	cholecystokinin receptor type A-like	1
	NW_017852949.1	LOC109608325	pyroglutamylated RFamide peptide receptor-like	1
	NW_017854760.1	LOC109607415	gamma-aminobutyric acid receptor subunit beta-like	1
	NW_017853630.1	LOC109605092	diuretic hormone receptor-like	1
	NW_017853958.1	LOC109606207	calcitonin gene-related peptide type 1 receptor-like	1
Hedgehog signaling	NW_017853413.1	LOC109603362	protein roadkill	1
	NW_017852941.1	LOC109600605	protein smoothened	1
	NW_017853048.1	LOC109596897	cullin-3	1
	NW_017853079.1	LOC109597991	casein kinase I-like	1
	NW_017853089.1	LOC109598269	indian hedgehog B protein	2

*Some genes involved in multiple pathways
